# Multipotent Mesenchymal Stromal Cells for the Prophylaxis of Acute Graft-versus-Host Disease—A Phase II Study

**DOI:** 10.1155/2012/968213

**Published:** 2011-12-25

**Authors:** Larisa A. Kuzmina, Natalia A. Petinati, Elena N. Parovichnikova, Lidia S. Lubimova, Elena O. Gribanova, Tatjana V. Gaponova, Irina N. Shipounova, Oxana A. Zhironkina, Alexey E. Bigildeev, Daria A. Svinareva, Nina J. Drize, Valery G. Savchenko

**Affiliations:** FGBU Hematology Research Center, Russia Fedration Ministry of Public Health, 125167 Moscow, Russia

## Abstract

The efficacy and the safety of the administration of multipotent mesenchymal stromal cells (MMSCs) for acute graft-versus-host disease (aGVHD) prophylaxis following allogeneic hematopoietic cell transplantation (HSCT) were studied. This prospective clinical trial was based on the random patient allocation to the following two groups receiving (1) standard GVHD prophylaxis and (2) standard GVHD prophylaxis combined with MMSCs infusion. Bone marrow MMSCs from hematopoietic stem cell donors were cultured and administered to the recipients at doses of 0.9–1.3 × 10^6^/kg when the blood counts indicated recovery. aGVHD of stage II–IV developed in 38.9% and 5.3% of patients in group 1 and group 2, respectively, (*P* = 0.002). There were no differences in the graft rejection rates, chronic GVHD development, or infectious complications. Overall mortality was 16.7% for patients in group 1 and 5.3% for patients in group 2. The efficacy and the safety of MMSC administration for aGVHD prophylaxis were demonstrated in this study.

## 1. Introduction

Severe graft-versus-host disease (GVHD) is a life-threatening complication following allogeneic hematopoietic stem cell transplantation (allo-HSCT) [1, 2]. Steroids are the first-line treatment for established GVHD and have a response rate of 30–50%. However, the outcome for patients with severe, steroid-resistant acute GVHD is poor, and overall survival is low [3]. A large variety of drugs, such as corticosteroids, methotrexate, cyclosporine, and mycophenolate mofetil, are used for GVHD prophylaxis, but, nevertheless, approximately 20–80% of patients develop GVHD after allo-HSCT [4, 5]. Therefore, it is very important to develop new, effective methods for GVHD prevention.

Multiple immune processes underlie the condition that is clinically expressed as GVHD after allo-HSCT [6]. The recipient's antigen-presenting cells play an essential role in GVHD development. Host dendritic cells (DCs) have been identified as crucial for the priming of the CD4+ and CD8+ donor T-cells that lead to GVHD onset [7] (“direct” allorecognition), while donor DC also participate through “indirect” allorecognition [8].

Bone-marrow-derived multipotent mesenchymal stromal cells (MMSCs) are able to differentiate *in vitro* into cells of mesenchymal origin [9, 10]. MMSCs are immunosuppressive, which has been demonstrated by *in vitro* coculture experiments with allogeneic lymphocytes. These cells do not induce lymphocyte proliferation, interferon-*γ* production, or the upregulation of activation markers [11, 12]. Several key mechanisms have been described that contribute to the MMSCs' direct or indirect alteration of T-, NK, B- and dendritic cell function.

The development of GVHD is mainly mediated by T-cells, and MMSCs can inhibit T-cell function. MMSCs downregulate the responses of naive and memory antigen-specific T-cells to their cognate peptides, and this is an effect that is contact dependent and does not appear to be mediated by DCs [13]. MMSCs are able to attenuate T-cell production of IL-2, which results in decreased formation of cytotoxic CD8+ T-cells [11] and directly inhibits NK cell proliferation and cytotoxic activity [14]. MMSCs cause the arrest of T-cell division, but they have no effect on early activation [15]. MMSCs induce apoptosis in activated T-cells but have no effect on resting T-cell proliferation [16]. Moreover, MMSCs promote the formation of Th1 and Th3 regulatory T-cells as well as IL-10 production, which both prevent GVHD development [17]. Studies of the interaction between MMSCs and B-cells have demonstrated that MMSCs can inhibit B-cell proliferation, differentiation, and chemotaxis [18, 19]. It is worth noting that MMSCs inhibit the production of antibodies, which makes MMSCs useful for treating autoimmune diseases, such as diabetes, arthritis, multiple sclerosis, and Crohn's disease [20].

MMSCs affect DCs, and this can alter their role as mediators of GVHD. MMSCs are capable of blocking the differentiation of monocytes and bone marrow precursors into DCs [21–23] and inhibiting the upregulation of CD1a, CD40, CD80, CD86, and HLA-DR expression during DC maturation, which maintains DCs in an immature state [24]. Moreover, MMSCs downregulate the secretion of the Th1-promoting cytokine IL-12 [24]. The generation of regulatory DCs may be mediated by soluble factors such as IL6 and prostaglandin E2 [25–27]. MMSCs also produce the “tolerogenic” cytokine IL-10 [28]. Thus, MMSCs help to prevent GVHD.

The ability of MMSCs to inhibit the development of GVHD requires not only cell-contact-dependent signals but also contact-independent signals, including prostaglandin E_2_, IL-6, IL-10, indoleamine 2,3-dioxygenase (IDO1), and transforming growth factor-*β* [28–31]. Of these, IDO1 in particular has been identified as a key mediator of MMSCs-based immunosuppression [32–34]. MMSCs inhibit complement activation by their production of factor H, and this may be an additional mechanism underlying the broad immunosuppressive capabilities of MMSCs [35].

Thus, there is sufficient *in vitro* evidence to support the use of MMSCs in the prevention and treatment of GVHD. Furthermore, a number of patient cohorts treated with MMSCs have been reported, and the results have been promising to date [36, 37]. No patients have had side effects during or immediately after the infusions of MMSCs [38].

It has been shown that umbilical cord blood-derived MMSCs were very effective for GVHD prevention but not for treatment in the xenogenic model of NOD/SCID mice [39, 40].

However, there are no clear published data regarding the preferred dose, the timing, and the frequency of MMSC infusion. A phase III, randomized controlled trial on the use of MMSCs in acute GVHD in humans is currently underway, and the first results are promising [41]. Importantly, neither acute nor long-term adverse events have been reported following the infusion of MMSCs, so it is possible to use these cells for aGVHD prevention.

The aim of this study was to investigate the safety and the efficacy of MMSC administration for GVHD prophylaxis. The randomized, prospective clinical trial was approved by the local ethics committee and was begun in October 2008. It was based on the random allocation of patients to the following two groups: (1) the group receiving the standard GVHD prophylaxis and (2) the group receiving the standard GVHD prophylaxis combined with the infusion of the hematopoietic stem cell donors' MMSCs. The data obtained demonstrated a significantly reduced development of aGVHD in patients who received MMSCs.

## 2. Materials and Methods

### 2.1. Patients

Thirty-seven patients who had received allo-HSCT from related donors were eligible for the study between October 2008 and May 2011. They were randomly allocated to the following two groups: (1) a group receiving the standard GVHD prophylaxis and (2) a group receiving the same prophylaxis combined with MMSC infusion. For each case, the MMSCs were derived from the corresponding hematopoietic stem cell donor. The patients' characteristics are presented in Table 1. All work was conducted in accordance with the Declaration of Helsinki (1964). This study was approved by the local ethics committee, and the donors and patients provided written informed consent.

### 2.2. Procedures and Definitions

The patients received either myeloablative or reduced-intensity conditioning (Table 1). Conditioning was myeloablative in 27 patients and included cyclophosphamide (60 mg/kg/day for 2 days) combined mainly with busulfan (4 mg/kg/day for 4 days). Ten patients had low-intensity conditioning regimens with either fludarabine phosphate (30 mg/m^2^/day for 6 days) combined with busulfan (4 mg/kg/day for 2 days) and antithymocitic globulin (ATG) (10 mg/kg/day for 4 days) or fludarabine phosphate (30 mg/m^2^/day for 5 days) combined with BCNU (200 mg/m^2^/day for 2 days), melphalan (140 mg/m^2^/day for 1 day), and ATG (20 mg/kg/day for 2 days). 

As GVHD prophylaxis patients received cyclosporine combined with methotrexate, some patients additionally received mycophenolate mofetil or prednisolone. 

Acute GVHD was graded according to internationally accepted criteria [42]. 

### 2.3. Laboratory Methods

The characteristics of the donors and the grafts are shown in Table 2. 

MMSCs were derived from 25–30 mL of the stem cell donors' bone marrow. For mononuclear cells, the bone marrow was mixed with an equal volume of alfa-MEM (ICN) media containing 0.2% methylcellulose (1500 cP, Sigma-Aldrich). After 40 min, most erythrocytes and granulocytes had precipitated, while the mononuclear cells remained in suspension. The suspended (upper) fraction was aspirated and centrifuged for 10 minutes at 450 g. 

The cells from the sediment were resuspended in a standard cultivation medium that was composed of alfa-MEM supplemented with 4% platelet lysate obtained from the donors' thrombocyte concentrates, as previously described [43], 2 mM L-glutamine (ICN), 100 U/mL penicillin (Ferein), and 50 *μ*g/mL streptomycin (Ferein). The cells were cultured at 27 × 10^6^ cells per T175 cm^2^ culture flask (Corning-Costar). When a confluent monolayer of cells had formed, the cells were washed with 0.02% EDTA (ICN) in a physiologic solution (Sigma-Aldrich) and then trypsinised (ICN). The cells were seeded at 4 × 10^3^ cells per cm^2^ of flask area. The cultures were maintained in a hypoxic atmosphere at 37°C in 5% CO_2_ and 5% O_2_. The number of harvested cells was counted directly; cell viability was checked by trypan blue dye exclusion staining. MMSCs were harvested in 6% polyglucin (public corporation Biochimik) and were either cryopreserved in 10% dimethyl sulphoxide (ROTH) or resuspended at a final concentration of 3–7 × 10^6^ cells per mL polyglucin, according to local guidelines, and infused intravenously into the patient at target dose 10^6^ per kg of body weight. 

All MMSCs were immunophenotyped with following markers: CD105, CD73, CD45, CD34, CD14, and HLA-DR using standard protocols. Antibodies were purchased at BD Pharmingen (CD105, CD59, CD73, CD90, CD31, CD34, and CD14), Sigma (CD45, FSP), and DAKO (HLA-DR). 

Total RNA was extracted from MMSCs by the standard method [44] and cDNA was synthesized using oligo(dT) primers. The gene expression level was quantified by real-time quantitative PCR using hydrolysis probes (Taqman) and ABI Prism 7000 (Applied Biosystems). Gene-specific primers were designed by the authors and synthesized by Syntol R&D. All primers and probes could be provided upon request. The relative gene expression level was determined by normalizing the expression of each target gene to that of *β*-actin and GAPDH and was calculated using the ΔΔ*C*_*t*_ method [45] for each MMSCs sample.

The criteria for the admission of MMSCs for clinical use included a spindle-shape morphology, the absence of visible clumps or contamination by pathogens, standard immune phenotyping [46] for the expression of surface molecules [47] and data on the *in vitro* differentiation of the cells into osteoblasts or adipocytes [48]. The cells were given as intravenous infusions when the blood counts were indicative of recovery following allo-HSCT (more than 1 × 10^9^/L leukocytes). The MMSC dose varied from 0.9 to 1.3 × 10^6^/kg. Cells for 7 of the infusions were harvested fresh from cultures and were given to the patients. For the other 11 cases, frozen cells were thawed and infused. 

### 2.4. Statistical Analysis

Data were analyzed using Student's *t*-test, with the last data collection in June 2011.

## 3. Results and Discussion

The MMSCs were expanded using the platelet lysate obtained from the donors' thrombocyte concentrates to avoid the transmission of zoonoses and the immune reactions possible if fetal calf serum were used [49]. All of the human components used for MMSC cultivation were from hematopoietic stem cell donors. It was assumed that MMSCs were transplantable across major histocompatibility complex class 1 barriers [41, 50]. However, it was recently shown that MMSCs are weakly immunogenic *in vivo* when transplanted across major histocompatibility complex class 1 barriers [51]. Thus, only MMSCs derived from the stem cell donors were used in this trial. The MMSC characteristics are presented in Table 2. Nineteen patients received MMSCs for GVHD prophylaxis. The exact date of infusion of MMSCs after transplantation, the MMSCs dose, and the exact pharmacologic immunosuppression applied in each patient are presented in the Table 3.

MMSCs were administered when the blood counts were indicative of leukocytes' recovery (leukocytes more than 1 × 10^9^ per liter). The time of administration was chosen at the time of graft activation and thus also at the time of GVHD manifestation. The median day of administration was day +28 after HSCT (19–54 days). Most of the patients had moderate fever and chills for 24 hours after MMSC administration, but there were no other complications. 

In the group receiving the MMSCs, acute GVHD of grade II developed in only one case (5.3%) (Tables 3 and 4). This case was a 59-year-old patient with CML, who had received a transplant in the 1st chronic phase from an HLA identical related donor. The blood counts were recovered at day +17 after allo-HSCT. The acute GVHD manifested at day +25 with skin involvement prior to MMSC injection. The MMSCs were administered only at day +30, as the required cell dose was not ready at day +17 due to the slow growth of the donor's MMSCs. The GVHD prophylaxis included cyclosporin, methotrexate, and prednisolone. The hematopoietic stem cell donor was 56-year-old, and his MMSCs grew slower than the MMSCs from other donors. Moreover, the relative expression level of the immunomodulatory factors expressed by his MMSCs was altered compared with the others (Table 2), the IL-6 level increased 2.7-fold, and the CSF1 level increased 1.8-fold, while the expression level of IL-10 decreased 1.7-fold, the CFH level decreased 12-fold, and the Ptges level decreased 11-fold. It is possible that the increased level of IL-6 led to the activation of the donor T-cells and B-cells [52]. Additionally, the increased level of CSF1 in the donors' MMSCs could have further enhanced macrophage activation, which would result in GVHD progression instead of inhibition. Moreover, the decreased production of factors that inhibit GVHD [26, 28–30] by MMSCs from this donor did not permit GVHD prevention. However, this single case of ineffective prophylaxis did not allow clear conclusions to be made about the significance of these factors expressed by MMSCs *in vitro* in the efficiency of GVHD prophylaxis. Nevertheless, clinical improvement was registered following MMSCs infusion, but, in one month, GVHD progression to grades III-IV and involving the skin, gut, and liver occurred. 

In the control group, 6 out of 19 patients had acute GVHD of grades II–IV (33.3%), which corresponded to the data from other investigators [2]. The outcomes of patients in each group are depicted in Table 4. 

Though the groups of patients are not great, yet there is a significant difference in the development of acute GVHD in patients who received MMSCs prophylaxis compared with the control group (*P* = 0.009). Despite the high statistical differences between these groups, the data could not provide solid evidence for the efficacy of the approach due to limited number of patients included in the trial. MMSC injection did not influence the development of chronic GVHD (Tables 3 and 4). The diagnosis of chronic GVHD is usually made earlier than 100 days after allo-HSCT [53]. The MMSCs injected at 28 days after allo-HSCT have only a small influence on chronic GVHD development likely due to their short life span and improper homing in the host [54, 55]. Clinical studies have shown that patients who develop GVHD have a lower risk of relapse [56]; moreover, it was shown that cotransplantation of mesenchymal stromal cells and hematopoietic stem cells may prevent GVHD, but the relapse rate was obviously higher than the control group [57], although we found no difference in the relapse rates of both groups of patients. It deserves to note that in this study MMSCs were not cotransplanted with hematopoietic stem cells but infused after transplant activation. 

There were no differences in the graft rejection rates or the infectious complications. The overall mortality was 22.2% in the standard prophylaxis group and 5.3% in the MMSC-treated group. 

## 4. Conclusions 

The current study is the first clinical trial to evaluate the feasibility and the safety of platelet lysate *in vitro* expanded stem-cell donor MMSCs for the prevention of acute GVHD. A high efficacy of MMSCs in GVHD prophylaxis was clearly demonstrated even on such limited number of patients, and no adverse events could be directly attributed to MMSC administration. In order to make a MMSC administration in the prevention of acute GVHD a candidate for inclusion in the standard protocols for GVHD prophylaxis, further investigations on the enlarged groups of patients should be performed. The data obtained support the development of new trials focused on the use of this approach in haploidentical and unrelated HSCT. 

## Figures and Tables

**Table 1:** Characteristics of the patients and treatments.

Group characteristics	First group (1)	Second group (2)
	Standard GVHD prophylaxis	Standard GVHD prophylaxis + MMSCs
Sex of patient, male/female	7/11	8/11

Median age, years (range)	29 (19–60)	34 (20–63)

Diagnosis, *n *		
AML/MDS	10	14
ALL	4	2
CML	3	3
CLL	1	

Disease stage, *n *		
complete remission	15	19
non-complete remission	3	0

Conditioning regimen, *n *		
RIC	4	6
MAC	14	13

Observation time, months	3.5–30.5	2.5–32

AML: acute myeloid leukemia, MDS: myelodysplastic syndrome, ALL: acute lymphoid leukemia, CML: chronic myeloid leukemia, CLL: chronic lymphoid leukemia, RIC: reduced intensity conditioning, MAC: myeloablative conditioning.

**Table 2:** MMSC donor and graft characteristics.

Donors	Values
Sex of donors, M/F	19/18

Median age, years (range)	34 (13–68)

MMSCs	

Culture passage at MMSCs harvest	0–3

Immunophenotype	
CD105 (Endoglin)	98,6 ± 0,2%
CD73 (SH3, SH4)	98,1 ± 0,5%
CD90 (Thy-1)	98 ± 0,5%
CD59	98,8 ± 0,1%
Fibroblast Surface Protein (FSP)	97 ± 0,4%
CD31 (PECAM-1)	2,5 ± 0,7%
HLA-DR	3,7 ± 0,7%
CD34	0,00%
CD45	4,5 ± 0,8%
CD14	2,0 ± 0,6%

Proportion of viable cells, %	95.3 ± 1.3%

Median MMSCs cell dose (×10^6^/kg, range)	1.1 (0.9–1.3)

Relative expression level of several genes in MMSCs on passage 2	
IL-6	2.57 ± 0.98
Ptges	10.07 ± 3.16
CSF1	2.04 ± 0.39
IDO1	0.36 ± 0.132
IL-10	2.73 ± 0.6
CFH	1.98 ± 0.36

**Table 3:** Patients treatment.

Patient	Age	Diagnosis	Conditioning regimen	MMSCs infusion				GVHD prophylaxis	GVHD stage (days after allo-HSCT)	Chronic GVHD
				Days after allo-HSCT	Passage number (P)	Dose per kg	Cryopreservation			
First group (1) standard GVHD prophylaxis										
KO	38	CML	MAC					CSA + Mtx + pr	II (15)	Yes
IV	27	ALL	MAC					CSA + Mtx+	II (62)	Yes
TB	19	AML	MAC					CSA + Mtx+	II(20)	Yes
CT	59	AML	RIC1					CSA + Mtx + MM	IIII–IV (100)	Yes
SV	25	AML	MAC					CSA + Mtx+	No	No
ZL	34	CML	MAC					CSA + Mtx + pr	No	No
RA	19	ALL	MAC					CSA + Mtx+	I (23)	No
KL	38	MDS	MAC					CSA + Mtx+	No	No
AD	38	AML	MAC					CSA + Mtx + MM + pr	No	No
SO	24	AML	MAC					CSA + Mtx+	I (26)	No
SE	51	AML	RIC2					CSA + Mtx+	II (39)	Yes
SI	20	AML	MAC					CSA + Mtx	II (10)	Yes
ZC	24	CML	MAC					CSA + Mtx	No	No
RA	31	AML	MAC					CSA + Mtx	I (36)	No
GN	60	AML	RIC2					CSA + Mtx	No	No
GJ	24	MDS	RIC2					CSA + Mtx	No	No
SN	36	ALL	MAC					CSA + Mtx	I (19)	No
PE	22	CLL	MAC					CSA + Mtx	No	No

Second group (2) standard GVHD prophylaxis + MMSCs										

AN	34	AML	MAC	+31	P2	1	No	CSA + Mtx	No	Yes
BT	20	CML	MAC	+28	P1	1,25	Yes	CSA + Mtx + pr	No	No
KA	22	ALL	MAC	+29	P1	1,1	Yes	CSA + Mtx	No	No
PS	29	AML	MAC	+31	P0 + P1	1	No	CSA + Mtx	No	No
PN	46	AML	MAC	+54	P3	1,08	Yes	CSA + Mtx	No	No
KS	37	MDS	RIC1	+28	P1	1,1	No	CSA + Mtx + MM	I (21)	No
SE	54	AML	RIC1	+50	P3	1,05	No	CSA + Mtx + MM	No	No
RS	47	MDS	RIC1	+34	P1	0,93	Yes	CSA + Mtx + MM	No	Yes
CA	44	AML	MAC	+32	P1	1,18	Yes	CSA + Mtx	No	No
TM	28	AML	MAC	+28	P2	1,05	Yes	CSA + Mtx	No	No
IL	63	AML	RIC1	+25	P1	0,9	No	CSA + Mtx + MM	No	No
CM	50	AML	RIC1	+26	P1	1,07	Yes	CSA + Mtx + MM	I (48)	No
BP	33	AML	MAC	+29	P1	1,15	No	CSA + Mtx	No	Yes
MK	33	AML	MAC	+22	P1 + P2	1,12	Yes	CSA + Mtx	I (17)	Yes
FE	39	CML	MAC	+24	P1 + P2	1,3	Yes	CSA + Mtx + pr	I (18)	No
TV	40	CML	MAC	+30	P1 + P2	1,26	Yes	CSA + Mtx + pr	II (25)	Yes
AI	22	ALL	MAC	+19	P0 + P1	1,25	No	CSA + Mtx	I (73)	No
DE	31	AML	RIC1	+28	P1	0,96	Yes	CSA + Mtx + MM	No	No
SS	34	AML	MAC	+24	P1 + P2	1,39	Yes	CSA + Mtx	No	No

AML: acute myeloid leukemia, MDS: myelodysplastic syndrome, ALL: acute lymphoid leukemia, CML: chronic myeloid leukemia, CLL: chronic lymphoid leukemia, RIC: reduced intensity conditioning ((1) fludarabine phosphate + busulfan + ATG, (2) fludarabine phosphate + BCNU + melphalan + ATG), MAC: myeloablative conditioning, CSA: cyclosporine, Mtx: methotrexate, pr: prednisolone, MM: mycophenolate mofetil.

**Table 4:** Patients' outcome.

Group characteristics	First group (1)	Second group (2)
	Standard GVHD prophylaxis (*n* = 18)	Standard GVHD prophylaxis + MMSC (*n* = 19)
Death at +100 days, *n*, %	1 (10%)	0
aGVHD (II–IV grade), *n*, %	6 (33.3%)	1 (5.3%)
cGVHD (lim + ext), *n*, %	6/17 (35.3%)	5/18 (27.8%)
Relapse rate, *n*, %	5/18 (27.7%)	4/19 (21.1%)
Alive, *n*, %	14 (77.7%)	18 (94.7%)

cGVHD form: lim-limited, ext-extensive.
